# The effect of COVID-19 on emergencies and pain among orthodontic patients attending a teaching hospital

**DOI:** 10.25122/jml-2022-0208

**Published:** 2022-10

**Authors:** Zahraa Mohammed Al-Fadhily, Dana Rifat Mohammed, Hala Adana Abdul Hammed, Akram Faisal Al-Huwaizi

**Affiliations:** 1 Department of Pedodontics, Orthodontics and Preventive Dentistry, College of Dentistry, University of Kufa, Najaf, Iraq; 2 Department of Pedodontics, Orthodontics and Preventive Dentistry, College of Dentistry, University of Basrah, Basrah, Iraq; 3 Ministry of Health, Baghdad, Iraq; 4 Department of Orthodontics, College of Dentistry, University of Baghdad, Baghdad, Iraq

**Keywords:** dentistry, orthodontics, COVID-19, orthodontic treatment, emergencies, pain, orthodontic patients

## Abstract

This study aimed to evaluate the effect of the COVID-19 outbreak on emergencies and pain among orthodontic patients attending a teaching hospital. The study was conducted among orthodontic patients receiving active orthodontic treatment or in a retention period at the College of Dentistry, University of Baghdad, Iraq. Their participation was voluntary, and they filled out an Arabic-translated questionnaire. The survey included general information, orthodontic problems, and a numerical rating scale for pain assessment. We used descriptive and inferential statistics (frequencies and intersecting frequencies), chi-square test and linear regression. Out of 75 orthodontic patients, only 54 (15 males and 39 females) were included in the study. The most encountered orthodontic problem was broken or movable bracket (55.6%), followed by long pocking wire 35.2%. In addition, 55.6% of the participants preferred to wait for the next appointment to see their orthodontist, and only 5.6% tried to treat the problem personally. There was no significant relationship between pain level, gender and age, whereas a strong significant association was found between pain intensity and orthodontic problems or emergencies. COVID-19 had a negative impact on orthodontic follow-up visits. The intensity of pain was strongly correlated with orthodontic problems or emergencies. Therefore, more attention should be given to patients, focusing on teaching them how to manage orthodontic emergencies during situations such as an outbreak.

## INTRODUCTION

Severe acute respiratory coronavirus initially started in Wuhan, China, in December 2019. The coronavirus disease 2019 (COVID-19) resulted from a new strain of the coronavirus [1]. This disease spreads through droplets and aerosols after coughing, sneezing, speaking, and contact with contaminated surfaces, with an incubation period of 2–14 days [2, 3]. There is an increased risk of COVID-19 cross-infection in dentistry [4, 5] due to the aerosols and splatters generated through the usual dental procedures and the physical closeness with the patient's face [6]. Orthodontics is a special branch of dentistry concerned with the management of different cases of malocclusion. Fixed orthodontic appliance is one of the most popular appliances in this field. Comprehensive therapy using fixed appliances takes about 2–3 years and usually requires continuous recheck appointments every 4–8 weeks. Delaying or postponing appointments until the end of the pandemic would unwittingly increase the treatment duration and result in other treatment consequences [7]. During the COVID-19 pandemic, it was recommended for patients to initially contact the orthodontist by telephone or online to evaluate their condition, assess the risk of COVID-19, give any temporary self-care advice, and plan an appointment if necessary [6].

Almost all orthodontic emergencies can be treated with advice from appropriate healthcare specialists, so only urgent cases should be given an urgent appointment [8]. During this emergency appointment, it is advised to avoid procedures that could generate aerosols. The therapy should be minimal, limited to trimming, cutting and wire adjustment, using appropriate pliers or distal end cutter to prevent trauma. Orthodontists can also use forceps or remove uncomplicated deboned orthodontic appliance components [9]. The effect of COVID-19 may not be restricted only to lost visits and fear of spreading the virus but also to the patient's psychology, emotion, and financial consequences [10].

This study aimed to evaluate the effect of the COVID-19 outbreak in terms of emergencies and pain among orthodontic patients attending a teaching hospital. Furthermore, we aimed to develop orthodontic treatment recommendations for patients during emergencies.

## Material and methods

This descriptive cross-sectional study included patients undergoing orthodontic treatment (active treatment or retention period) at the orthodontic department, College of Dentistry, University of Baghdad, Iraq, from the start of the COVID-19 pandemic until starting this study, Jan 2022. The study was carried out for three weeks using a printed questionnaire. Out of the 75 orthodontic patients, only 54 (15 males and 39 females) were included in the study, depending on their orthodontic treatment duration. The questionnaire was translated into Arabic and divided into three sections. The first section included a description of the purpose of the study, information on voluntary participation without identification of any personal information, and a confirmation from the participants that they are in active treatment or retention period. The second section included information regarding age and gender, type of appliance, treatment duration and problems encountered during the pandemic. Finally, the third section included the numerical rating scale for pain, a segmented numerical scale with numbers from 0 to 10, with 0 representing no pain and 10 representing the worst pain imaginable [11]. The higher the score, the greater the pain, with no different measures among different ages of orthodontic patients, as shown in appendix 1.

### Statistical analysis

We used descriptive statistics represented by frequencies and intersecting frequencies. The Chi-square test was used to assess the relationship between variables and linear regression to study the effect of the pandemic. The level of statistical significance was set at P<0.05. All analyses were performed using the statistical software program Statistical Package for the Social Sciences (SPSS) (IBM Company, Chicago, USA, Version 19).

## Results

The total sample included 54 patients (72.2% females and 27.8% males) with an average age of 18.8 years. 40.6% of participants were ≤17 years, 44.4% were in the 18–23 age range, and 15% were ≥24. Most participants (35.2%) were following treatment for 13–24 months, followed by the ≥48 months category (25.9%) and the 25–36 months category (20.4%). 81.5% of the individuals used fixed orthodontic appliances, while 11.1% used removable ones. The participants' characteristics are presented in Table 1.

**Table 1:** Sample characteristics.

Variables		Frequencies	Percentage
**Gender**	Males	15	27.8
	Females	39	72.2
**Age (years)**	≤17	22	40.6
	18–23	24	44.4
	≥24	8	15
**Treatment duration (months)**	1–12	8	14.8
	13–24	19	35.2
	25–36	11	20.4
	37–48	2	3.7
	≥48	14	25.9
**Type of orthodontic appliance**	Removable	6	11.1
	Fixed	44	81.5
	Others	4	7.4

**Figure 1:**
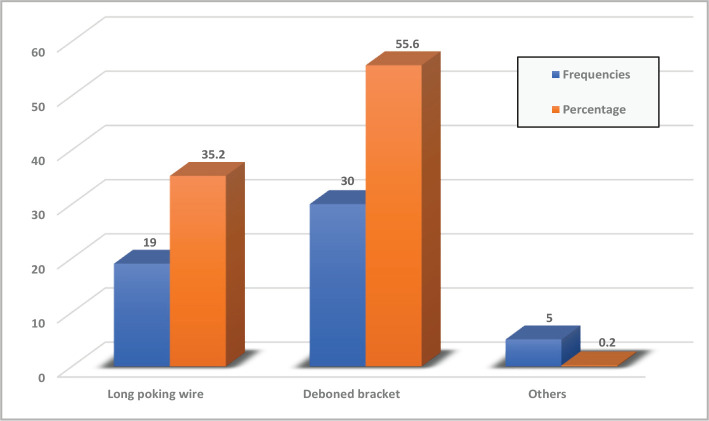
Frequencies and percentages of orthodontic emergencies.

Regarding emergencies, 55.6% of participants mentioned that they suffered from broken or removable brackets, and 35.2% suffered from long pocking wire (Figure 1).

**Figure 2:**
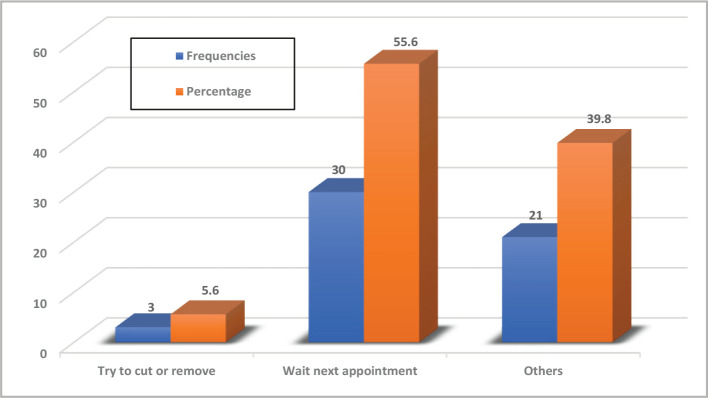
Frequencies and percentages of procedures done to resolve the emergency.

55.6% of the participants who suffered from orthodontic problems said they preferred waiting for the next appointment to see their orthodontist. In comparison, 5.6% tried to treat the problem personally, while 39.8% did not have a problem (Figure 2).

The numerical rating pain scale results showed that 27.8% suffered from moderate pain due to orthodontic problems, 24.1% suffered from mild pain, and 24.1% of individuals had no pain. However, 14.8% had severe pain, and 9.3% had the most severe pain (Table 2).

**Table 2:** Frequencies and percentages of numerical rating pain scale.

Type	N	%
No pain	13	24.1
Mild	13	24.1
Moderate	15	27.8
Severe	8	14.8
Most severe	5	9.3
Total	54	100

Most males (11.11%) felt mild pain, and 3.7% felt the most severe pain. With respect to females, 22.22% had moderate pain, and 1.85% had the most severe pain. There was no relationship between gender and pain intensity (p>0.05) (Table 3).

**Table 3:** Pain scale – gender relation.

Gender/pain	Male		Female		Total	
	N	%	N	%	N	%
**No pain**	4	7.42	11	20.37	15	27.79
**Mild**	6	11.11	7	12.96	13	24.07
**Moderate**	3	5.56	12	22.22	15	27.78
**Severe**	2	3,7	6	11.11	8	14.81
**Most severe**	2	3,7	1	1.85	3	5.55
**Total**	17	64.79	37	68.51	54	100

Chi-square test; P-value>0.05.

Table 4 shows a convergence among the age groups in terms of pain severity. Most participants who experienced moderate pain were in the adolescent (12–17 yrs.) (12.96%) and the youth groups (18–23 yrs.) (12.96%). The lowest percentage (1.85%) for those who felt the most severe pain was among adolescents between 12 and 17 years. There was no significant relationship between age and intensity of feeling pain (p>0.05). We analysed the effects of an orthodontic problem on the severity of pain using linear regression.

**Table 4:** Pain scale –age relation.

Age/pain	12–17		18–23		More than 24		Total	
	N	%	N	%	N	%	N	%
**No pain**	6	11.11	4	7.4	2	3.7	12	22.21
**Mild**	4	7.4	7	12.96	2	3.7	13	24.06
**Moderate**	7	12.96	6	11.11	4	7.4	17	31.07
**Severe**	2	3.7	3	5.55	1	1.85	6	11.1
**Most severe**	1	1.85	2	3.7	3	5.55	6	11.1
**Total**	20	37.02	22	40.72	13	16.65	54	100

Chi-square test; P-value>0.05.

The calculated F value was 6,663 with a significant level (p=0.000), revealing a strong and positive effect of having an orthodontic problem on the severity of the pain (β=0.556). It means that a change in one unit in the orthodontic problem would lead to a change in the severity of pain of 0.556. The coefficient of determination (R2) was 0.225, meaning that the orthodontic problem explained 22.5% of the variance that occurred in the severity of pain and that 77.5% resulted from 22.5–100%, which is an explanatory variance from factors not included in the regression model (Table 5).

**Table 5:** The effects of an orthodontic problem on the severity of pain.

Constant limit (a)	Beta coefficient (β)	Coefficient of determination (R^2^)	Calculated value (F)	Calculated value (T)	Significance	The decision
**2.944**	0.556	0.225	6.663	5.698	0.000	There is an effect

## Discussion

The COVID-19 outbreak imposed many limitations on our daily life since stay-at-home and quarantine were ordered in many countries to reduce the spread of the virus, an essential step in eliminating or reducing the worldwide impact. Additionally, special measures were applied in dental clinics to reduce aerosols during procedures, such as implementing Teledentistry, where dentists can use information and technology to assist and advise patients, minimising anxiety about treatment duration, support good outcomes and oral health care, or using particular materials to reduce environmental contamination [12–14]. Orthodontic treatment is a procedure requiring follow-up visits for active adjustments and may be negatively affected by delays and/or missing appointments. It is obvious that the patients and the entire dental team are at great risk of being infected; therefore, regarding orthodontic follow-up visits, only emergency orthodontic treatment should be carried out. Additionally, the term emergency in orthodontics is not clearly understood, and it can refer to any unscheduled appointment that the orthodontic appliance may require to relieve discomfort and ensure treatment continuity [15]. Most participants in this study were females (72.2%) compared to males (27.8%) since females are more concerned about their appearance and aesthetics, which agrees with Turkistani [16], who found that females sought orthodontic treatment more often than males. The age of the patients was mostly below 24 years, supporting other research that most orthodontic patients are adolescents and young adults [17–19]. Orthodontic patients are more concerned about any increase in duration since delay or missing orthodontic appointments may prolong the duration of treatment by 1.09 months for each missed follow up-visit [20, 21]. In our study, 35.2% of participants underwent orthodontic treatment for 13–24 months, followed by 25.9% for more than 48 months. Most participants had fixed orthodontic appliances (81.5%), which can be explained by the fact that it is considered the most comprehensive approach used for treating most malocclusions [17–19, 22].

In this study, we introduced a list of common orthodontic problems that may occur during orthodontic treatment. Most participants suffered from broken or movable brackets (55.6%), the most reported problem in the orthodontic literature [14, 15, 23], which could be due to poor oral hygiene, improper diet or defective bonding procedures. The second problem that mostly occurred was long pocking wire (35.2%) which is one of the common problems during the alignment and levelling phase of fixed orthodontic treatment [24]. Regarding the management of orthodontic emergencies undertaken by the participants, 55.6% preferred to wait for the next appointment to see their orthodontist, which may be related to the fear of getting infected with the virus from other dental offices. Only 5.6% of the participants tried to treat the problem personally by cutting the wire and/or removing the broken brackets. During orthodontic treatment, lighter forces are less traumatic and painful and are thought to be ideal, but there is a controversy regarding this recommendation, and extreme forces may be unavoidable even in the initial phases of treatment [19]. This may be related to our findings, in which 27.8% of the participants suffered from moderate pain and only 9.3% suffered from severe and the most severe pain, with no significant relationship with gender and age. The variation in pain perception could be related to an individual's biological variations to compensate for pain. When analysing the effect of an orthodontic problem on the severity of pain, there was a significant strong association since the long pocking wires and broken brackets were primarily associated with traumatic injuries to the inner side of the cheek or the mucosa and discomfort. Limitations of the study were the sample size and the duration of sample collection, which may affect generalising the results to all orthodontic patients at the teaching hospitals of the College of Dentistry, University of Baghdad. In addition, data were self-reported, so it depended on the patient's memory regarding the orthodontic problems and pain that may have influenced their answers. Finally, the study was based only on the Iraqi population, which may not give an idea about communities from other countries. Further research is required to study the situation in other regions, which may clarify the cross-cultural differences. Further investigations are needed to evaluate the awareness of orthodontists to deal with emergencies and the impact of delayed or missed orthodontic appointments during the COVID-19 outbreak on treatment outcomes.

## Conclusion

COVID-19 had a negative impact on orthodontic follow-up visits in this study. Most orthodontic patients in our sample suffered from problems related to their fixed orthodontic appliance, mainly broken brackets and long pocking wires, which required an emergency recall. The intensity of pain was strongly correlated with orthodontic problems; therefore, orthodontists should focus more on teaching their patients how to deal with orthodontic emergencies during situations such as an outbreak.

## Recommendations

Orthodontists should have an emergency plan for the management of their patients. In dealing with a pandemic of this nature, the following recommendations are suggested to be followed:

- All elective treatment, such as routine orthodontic treatment, should be postponed until it is permitted again after the end of the pandemic;
- Most orthodontic appliances can be left in situ for some months without negative effects if patients continue with the usual instructions of oral care, low sugar diet, and avoidance of hard, sticky foodstuffs which could break the wire or brackets;
- Orthodontists should guide patients and teach them how to manage minor orthodontic emergencies at home by providing a means of communication (phone number and\or email) to share any questions and problems related to their appliance;
- There should be a specialised emergency unit for urgent cases, following the necessary precautions and infection prevention and control protocol with guidelines issued by local health and regulatory authorities.

Since the most commonly encountered orthodontic emergencies in literature and our study were deboned brackets and long pocking wire, it is recommended to advise the patients to keep the bracket in its place if no pain or discomfort is experienced. If there is trauma, the orthodontist should teach the patient to remove it via video/photos. Additionally, providing the patient with orthodontic wax in case of long pocking thick and stiff wire, or using a nail clipper and/or scissor to cut the end of the small and light wire.

## Supplementary Material


