# Supplementary material for: The effect of COVID-19 on emergencies and pain among orthodontic patients attending a teaching hospital
Source: J Med Life. 2022 Oct;15(10):1267–71. doi: 10.25122/jml-2022-0208 (PMC9675302; doi:10.25122/jml-2022-0208)
Supplement: Supplementary file 1 [file JMedLife-15-1267-s001.pdf]

QUESTIONNAIRE FORM

Section 1

This questionnaire is carried out to study the impact of the COVID-19 outbreak on emergencies, disabilities and pain associated with orthodontic treatment.
Participation in this study is completely voluntary:

- Yes, I am willing to participate;
- No, I am not willing to participate;
- I confirm that I am in active orthodontic treatment or a retention period.

Section 2

- Age (date of birth) *
- Sex*
  a. Male;
  b. Female.
- Number of months in treatment*
  a. 1–12 months;
  b. 13–24 months;
  c. 25–36 months;
  d. 37–48 months;
  e. More than 48 months.
- Type of orthodontic treatment you are undergoing*
  a. Removable appliance;
  b. Fixed appliance;
  c. Other.

Orthodontic problem (illustrative image for simplification purposes)

Debonded bracket	Long poking wire	Sharp end of ligature tie
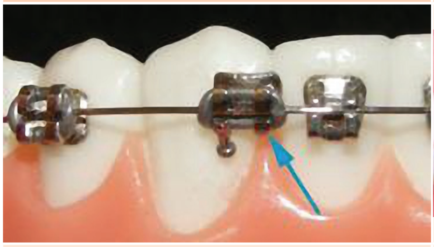	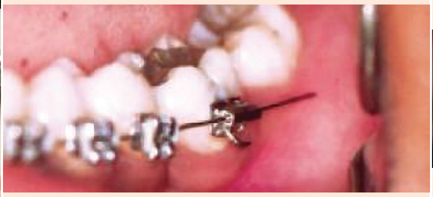	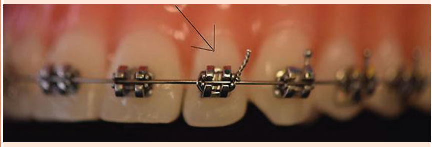
Try to remove;<br>Go to another clinician;<br>Wait for next appointment;<br>Other.	Try to cut;<br>Go to another clinician;<br>Wait next appointment;<br>Other.	Try to cut or bend;<br>Go to another clinician;<br>Wait next appointment;<br>Other.
**Broken fixed retainer**	**Lost/broken removable retainer**	**Broken or loose expander**
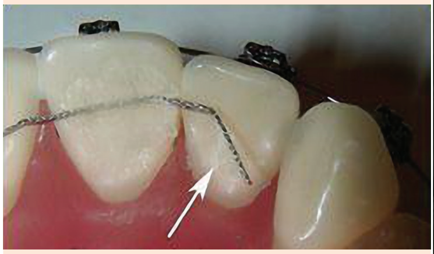	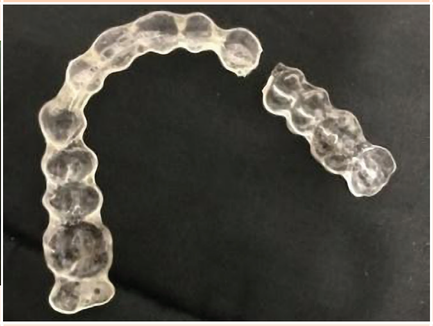	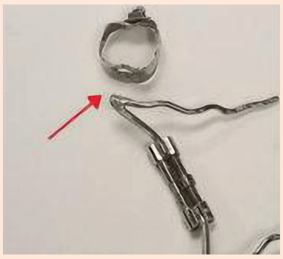
Try to repair;<br>Go to another clinician;<br>Wait next appointment;<br>Other.	Try to repair;<br>Go to another clinician;<br>Wait next appointment;<br>Other.	Try to repair;<br>Go to another clinician;<br>Wait next appointment;<br>Other.

Oral ulcer	Inflammation	Swelling
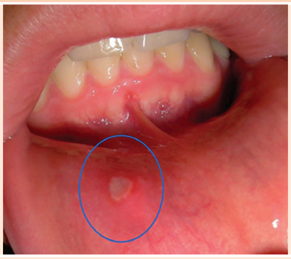	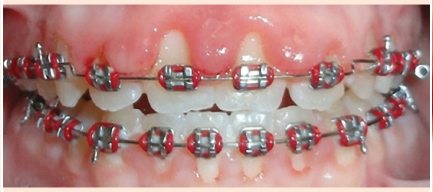	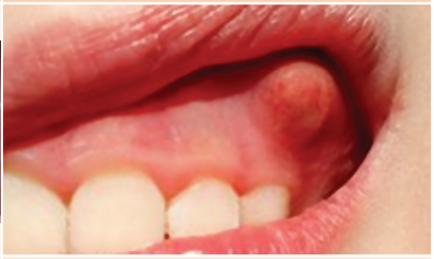
Take analgesic;<br>Take antibiotic;<br>Use mouth wash;<br>Go to another clinician;<br>Without treatment;<br>Other.	Take analgesic;<br>Take antibiotic;<br>Use mouth wash;<br>Go to another clinician;<br>Without treatment;<br>Other.	Take analgesic;<br>Take antibiotic;<br>Go to another clinician;<br>Without treatment;<br>Other.
**Loose TAD**	**Bleeding**	**Trauma from power chain**
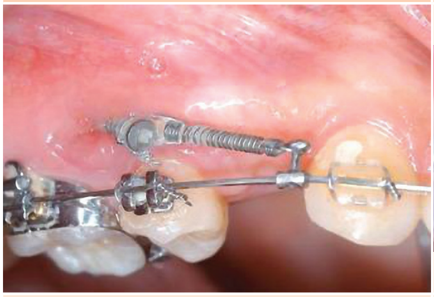	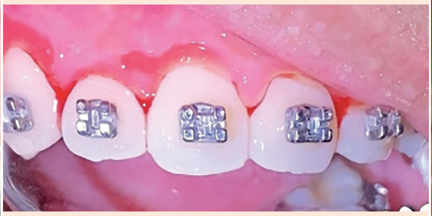	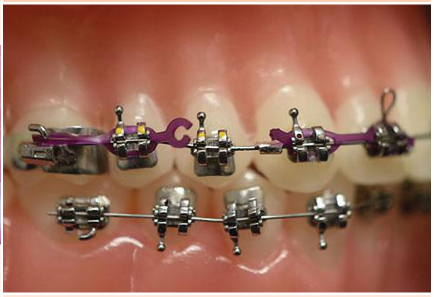
Try to remove it;<br>Without management;<br>Go to another clinician;<br>Wait next appointment;<br>Other.	Use mouthwash;<br>Go to another clinician;<br>Without treatment;<br>Other.	Try to remove it;<br>Without management;<br>Go to another clinician;<br>Wait next appointment;<br>Other.
**Pain**	**Others**	-
Take analgesic;<br>Go to another clinician;<br>Without treatment;<br>Other.	-	-

Section 3

Numerical Rating Pain Scale

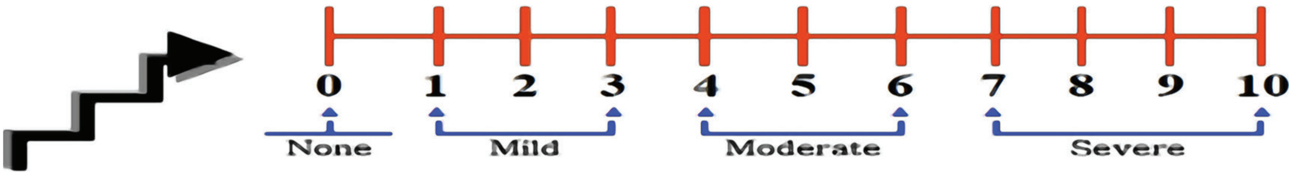

0: indicates the absence of pain; 10: represents the most intense pain possible.
